# Feasibility of robotic telesurgery over wired and private 5 G networks within Brazil’s public health system (SUS): a pilot study

**DOI:** 10.1016/j.clinsp.2026.100926

**Published:** 2026-04-02

**Authors:** Everson L.A. Artifon, Felipe Kfouri, Jose Pinhata Otoch, Luiz P. Kowalski, William Nahas, Gustavo Ebaid, Paulo Pego-Fernandes, Edmund C. Baracat, José Maria Soares Júnior, Ulysses Ribeiro, Marcos Samano, Ricardo Terra, Pedro Nabuco, Marco A.V. Kulcsar, Gabriel dos Anjos, Eduardo Motta, Ricardo Zugaib Abdalla, Sergio Damous, Alessandro Belon, Giovanni G. Cerri

**Affiliations:** a Departamento de Cirurgia, Faculdade de Medicina da Universidade de São Paulo (FMUSP), São Paulo, SP, Brazil; b Departamento de Cardiopneumologia, Faculdade de Medicina da Universidade de São Paulo (FMUSP), São Paulo, SP, Brazil; c Disciplina de Ginecologia, Departamento de Obstetrícia e Ginecologia, Hospital das Clínicas, Faculdade de Medicina da Universidade de São Paulo (HCFMUSP), São Paulo, SP, Brazil; d Departamento Gastroenterologia, Faculdade de Medicina da Universidade de São Paulo (FMUSP), São Paulo, SP, Brazil; e Departamento Radiologia e Oncologia, INOVA-HC, São Paulo, SP, Brazil

**Keywords:** Robotic surgery, Telesurgery, 5 G, Network Performance, Feasibility Study, Surgical Simulation

## Abstract

**Objective:**

To assess the feasibility and performance of a robotic telesurgery platform operating within the Brazilian Unified Health System (SUS) infrastructure in a dry lab environment under wired and private 5 G network conditions.

**Methods:**

Over three consecutive days, robotic surgeons performed three standardized dry lab tasks from a remote console at PROMIN‒FMUSP, with the robotic unit at HU-USP. Sessions were conducted using either a wired or a private 5 G network. Performance outcomes included task success and completion time. Connectivity metrics ‒ latency, jitter and packet loss ‒ were recorded; and per-participant mean values were used in descriptive analyses. Surgeons completed post-session assessments of perceived safety, usability, and mental workload.

**Results:**

Twenty-eight robotic surgeons completed dry lab sessions (23 wired, 5 private 5 G). Task success was 100% for Task 1, 82.1% for Task 2, and 89.3% for Task 3; all failures occurred in wired sessions. Median completion times were 26 s (Task 1), 4.5 min (Task 2), and 8.5 min (Task 3). Median latency and jitter were significantly higher in 5 G than in wired sessions (latency: 32.4 vs. 12.0 ms; jitter: 30.8 vs. 6.8 ms). Median packet loss was low under both conditions (0.0% in 5 G; 0.09% in wired). Most surgeons rated the system as safe (78.6% assigned the maximum safety score) and considered it suitable for high-complexity procedures (67.9%).

**Conclusion:**

Robotic telesurgery within SUS infrastructure was feasible in a simulated environment. Although the private 5 G network exhibited higher latency and jitter than the wired connection, the task completion and usability were preserved, supporting further evaluation in controlled clinical settings.

## Highlights

• Telesurgery was feasible over SUS infrastructure in a dry lab setting.
• Private 5 G delivered higher latency and jitter than wired connections.
• All 5 G sessions completed all tasks successfully.
• Surgeons rated safety and suitability for complex procedures highly.

## Introduction

Telesurgery expands access to specialized surgical care by allowing experts to operate robotic systems from remote locations.^1^^,^^2^ While advances in robotics and network infrastructure have enabled successful pilot procedures, reliable performance depends on low-latency, low-jitter connectivity and robust system stability.^2^ The Brazilian Unified Health System (*Sistema Único de Saúde* ‒ SUS) serves a large, geographically diverse population and could benefit from scalable remote surgical solutions. However, the feasibility of telesurgery within SUS infrastructure has not been systematically evaluated.

We conducted a dry lab feasibility study of a robotic telesurgery platform connecting two academic hospitals in São Paulo. The primary aim was to assess network performance and task completion under wired and private 5 G conditions. Secondary aims included characterizing surgeon perceptions and identifying operational considerations relevant to future clinical deployment.

## Materials and methods

### Study design and setting

This was a feasibility study conducted over three consecutive days to evaluate the performance of a robotic telesurgery platform using the SUS infrastructure in a simulated (dry lab) environment. Surgeons operated from the robotic console located at PROMIN‒FMUSP, while the robotic unit was positioned at *Hospital Universitário da Universidade de São Paulo* (HU-USP). Sessions were performed under two network configurations: 1) A wired connection and 2) A private 5 G network connection. Due to logistical constraints, the private 5 G network was available only during one study period (the afternoon session of Day 3), during which all tasks were performed exclusively using the 5 G connection. In all other study periods, tasks were performed using the wired connection. Surgeons were allocated to network conditions based on scheduling availability; no randomization or stratification was performed

### Robotic platform and experimental configuration

**Fig. 1:**
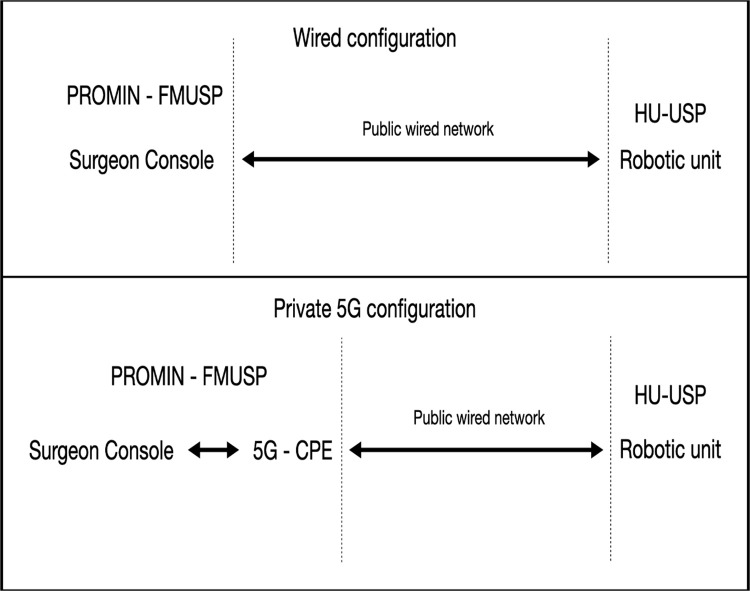
Network configurations.

A Toumai®/MicroPort® robotic surgery platform was used. The system consisted of a surgeon console installed at PROMIN‒FMUSP and a robotic unit installed at HU-USP. The robotic unit side was connected through the wired public network at all times. The console side was connected using either the public wired network or a private 5 G link, depending on the test condition (Fig. 1). In the 5 G configuration, the institutional wired connection served as backhaul to a 5 G Customer Premises Equipment (CPE) gateway. This arrangement introduced a wireless radio hop into the data path, reproducing the latency and jitter characteristics of a private 5 G segment while maintaining controlled backhaul conditions.

### Surgeon eligibility criteria

Surgeons were eligible to participate if they had prior experience in robotic surgery and had completed certification training, including demonstrated proficiency on a robotic surgery simulator.

### Tasks and dry lab models

**Fig. 2:**
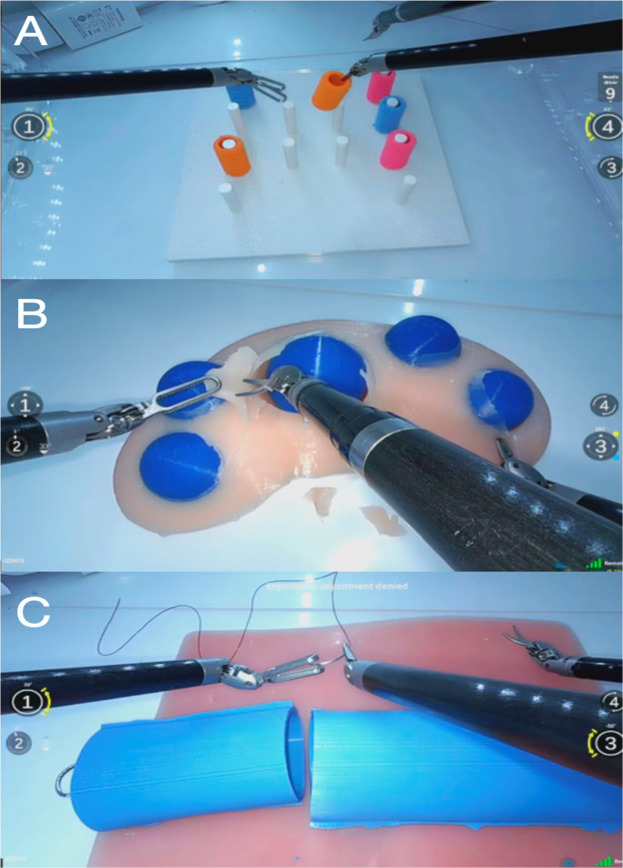
Surgical tasks. (A) Object manipulation. (B) Dissection. (C) Suturing.

Participants performed three standardized dry lab tasks designed to emulate fundamental surgical maneuvers, including: 1) Object manipulation, 2) Dissection, and 3) Suturing (Fig. 2). The tasks were meant to evaluate different surgical skills, respectively: assessing hand-eye coordination, tissue handling, and dexterity. Dry lab models and instrumentation were kept constant throughout the study. Predefined criteria for success, partial success and failure were applied to each task.

### Network performance metrics

Network quality was assessed continuously during each session using the robotic system's built-in connectivity indicators. Recorded metrics included latency, jitter, and packet loss. Measurements were sampled approximately every 5-minutes from the robotic system’s built-in connectivity monitor. This sampling interval may not capture transient latency or jitter spikes occurring during critical maneuvers. Per-participant mean values were used in descriptive analyses to provide a summary measure per session.

### Private 5 G configuration

In the private 5 G condition, the institutional wired connection served as backhaul to a 5 G CPE gateway at the console side. The study platform was the sole client on the private 5 G segment; no QoS shaping, or prioritization was configured or required.

### Task performance outcomes

For each task, the following performance outcomes were recorded: 1) Completion time and 2) Success, partial success or failure in accomplishing the predefined procedures. Failures were defined as the inability to complete the task within the allocated time or an interruption that prevented safe continuation of the maneuver. Partial success was defined as completion of the task within the allotted time but with minor technical errors that did not prevent achievement of the primary objective and would not be expected to compromise procedural safety. Examples included suboptimal tissue handling or, for suturing, an imperfect suture line or incomplete edge-to-edge approximation without loss of knot integrity.

Task outcomes were adjudicated by a designated surgeon observer present during all sessions, using the predefined criteria. No inter-rater reliability assessment was performed.

### Surgeon demographics, perception and mental workload

Surgeon characteristics were recorded using a structured questionnaire (age, sex, specialty/subspecialty, years of surgical and robotic experience, approximate number of robotic cases in the prior year, prior telesurgery experience, and familiarity with robotic platforms). After completing each session, surgeons provided a subjective assessment of system performance and usability under each network condition using 5-point Likert scales. Mental workload associated with task execution was assessed using an adapted NASA-TLX with five domains (mental demand, temporal demand, performance, effort, and frustration, while the physical domain was omitted).^3^ Additional post-session items addressed perceived video quality, latency, frame loss, audio quality and synchronization, control responsiveness, precision and dexterity compared with conventional robotic systems, perceived safety, communication effectiveness, and acceptability of the current latency for varying levels of procedure complexity. Binary and categorical items captured perceived loss of command or video, errors or near-miss events, and ability to recover control after a failure. Clinical acceptance items included suitability for clinical use, minimum technical requirements, main concerns, and suggested improvements.

### Data handling and analysis

Descriptive statistics were used to summarize network and performance measures. Continuous network metrics were compared between wired and private 5 G conditions using Mann-Whitney *U* tests; categorical task outcomes were compared using Fisher exact tests. Ordinal regression was used to explore associations between network type, connectivity metrics, robotic experience, and surgeon-reported outcomes.

For the primary analysis, partial successes were conservatively classified as failures. Only tasks completed without any technical deviation from the predefined criteria were counted as successful. This stricter criterion was adopted to ensure a rigorous assessment of feasibility and to avoid overestimating task performance in this pilot study.

Given the exploratory nature of this feasibility study and the small number of prespecified comparisons, no formal correction for multiple testing was applied. Analyses were performed in Python using Pandas and NumPy, with scikit-learn for PCA and stats models for ordinal regression; significance was set at *p* < 0.05.

### Ethics statement

This study was conducted in a simulated dry lab environment without direct patient involvement. Institutional approval was obtained as required by local regulations (approval date: November 14, 2025, IRB approval number: CAAE 93,291,425.7.0000.0076). All participating surgeons provided informed consent. The robotic platform was provided by the manufacturer at no cost for the purpose of this feasibility study. Clinical trial registration was not required for this study design.

## Results

### Participants and study sessions

A total of 28 robotic surgeons completed dry lab sessions over three consecutive days. Participant demographics are summarized in Table 1. Most sessions were conducted using a wired network connection (*n* = 23), whereas the private 5 G network was available only during the afternoon session of Day 3, resulting in a smaller number of sessions under 5 G conditions (*n* = 5).

**Table 1:** Participant characteristics.

Participant characteristics (n = 28)	
Age, years, median (IQR)	47.5 (42.8‒54.0)
Sex, n (%)	Male 25 (89.3), Female 3 (10.7)
Specialty, n (%)	General surgery 7 (25.0); Digestive surgery 4 (14.3); Head and neck surgery 4 (14.3); Thoracic surgery 4 (14.3); Urology 3 (10.7); Gynecology 3 (10.7); Plastic surgery 2 (7.1); Pediatric surgery 1 (3.6)
Surgical experience, years, median (IQR)	3.5 (2.0‒11.5)
Robotic procedures in prior year, median (IQR)	20 (3.8‒60)
Prior telementoring experience, n (%)	2 (7.1%)

### Surgeon perception and safety

Among the 28 participants, 4 (14.3%) reported some degree of loss of control during system use. Three of this reported full recovery of controls afterward. Only one participant (3.6%) reported errors or near-miss events during task execution. Overall acceptance of the platform was high: 27 of 28 participants (96.4%) considered the system suitable for clinical use, while one declined to answer this item (item non-response). Regarding perceived safety, ratings were predominantly at the highest level with 22/28 (78.6%) assigning a safety score of 5 out of 5 and 6/28 (21.4%) assigning a score of 4 out of 5. For procedural complexity, 19/28 (67.9%) believed the system could support high-complexity procedures and 9/28 (32.1%) considered it more suitable for medium-complexity procedures.

### Network performance

**Fig. 3:**
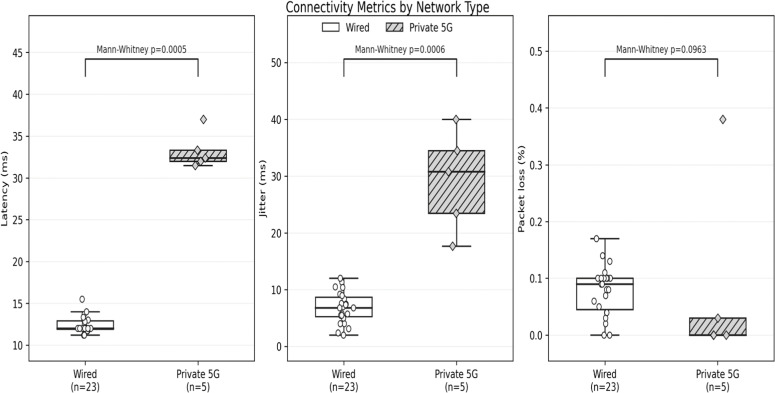
Connectivity metrics by network type.

Connectivity metrics were recorded across all sessions. Using per-participant mean values, wired sessions showed a median latency of 12.0 ms (IQR 11.9‒12.9; range 11.2‒15.5) and median jitter of 6.8 ms (IQR 5.25‒8.7; range 2.0‒12.0). Private 5 G sessions showed a median latency of 32.4 ms (IQR 32.0‒33.3; range 31.5‒37.0) and median jitter of 30.8 ms (IQR 23.5‒34.5; range 17.7‒40.0). Median packet loss was 0.09% (IQR 0.045‒0.10; range 0.0‒0.17) for wired sessions and 0.0% (IQR 0.0‒0.03; range 0.0‒0.38) for 5 G sessions. These values are clinically negligible and unlikely to affect surgical performance at the levels observed. Fig. 3 illustrates the differences observed. On nonparametric testing, latency and jitter were significantly higher in 5 G than in wired sessions (Mann-Whitney *p* = 0.0005 and *p* = 0.0006, respectively), while packet loss did not differ significantly (*p* = 0.096).

### Task performance outcomes

All participants completed Task 1 successfully (28/28, 100%). For Task 2, overall success was 23/28 (82.1%), with failures occurring only during wired sessions (5/23, 21.7%); all five 5 G sessions were completed successfully (5/5, 100%). For Task 3, overall success was 25/28 (89.3%), and partial successes occurred only during wired sessions (3/23, 13.0%); all 5 G sessions were completed successfully (5/5, 100%). Fisher exact tests did not show a significant difference in success rates between network types for Task 2 (*p* = 0.55) or Task 3 (*p* = 1.00). However, due to the non-randomized allocation and temporal confounding, this pattern should not be interpreted as evidence of differential performance by network type.

### Task completion times

Task completion times were recorded for all sessions. For Task 1, the median completion time was 26 s (IQR 20‒31.5 s). For Task 2, the median completion time was 4.5 min (IQR 3.0‒6.0). For Task 3, median completion time was 8.5 min (IQR 5.75‒10.25). When stratified by network type, completion times were comparable between wired and private 5 G sessions, although formal statistical comparison was limited by the small sample size in the 5 G group.

### Workload and perception outcomes

NASA-TLX subscale scores were summarized via PCA. PC1 explained 51.3% of the variance and PC2 explained 20.3% (cumulative 71.6%), indicating a dominant general workload component. Given the small sample size (*n* = 28) relative to the number of subscales (5), PCA results should be considered exploratory and interpreted with caution. Ordinal regression models for perceived safety and procedure complexity level showed no significant associations with network type (5 G vs. wired), mean latency, mean jitter, or robotic experience (all *p* ≥ 0.27).

## Discussion

This feasibility study demonstrates that robotic telesurgery tasks can be performed over SUS infrastructure in a simulated dry lab environment, with high task completion rates and favorable surgeon perceptions under both wired and private 5 G network conditions. The private 5 G segment introduced significantly higher latency (median 32.4 vs. 12.0 ms) and jitter (30.8 vs. 6.8 ms) compared with the wired connection, yet task success was preserved across all conditions and most surgeons rated the system as safe and suitable for clinical application. These findings provide initial evidence that telerobotic surgery within Brazil's public healthcare network can achieve acceptable performance parameters.

The median 5 G latency of 32.4 ms observed in our study falls below the delay levels shown to affect operability in experimental models; brain-activity-based assessments suggest that perceptible control degradation emerges beyond ∼150 ms, with subtle precision loss at lower thresholds.^4^ Clinically, multiple 5 G telesurgery studies report stable performance at latencies in the tens of milliseconds, including a prospective controlled telecholecystectomy trial, a multicenter phase I study across China using EDGE MP1000 systems, and a prospective single-arm remote hepatectomy using the MP1000.^5^^–^^7^ Toumai-based remote gastrectomy series and trials, along with multicentric experiences, report comparable latency profiles and successful completion rates.^8^, ^9^, ^10^ Feasibility has also been demonstrated across other specialties and configurations, including gynecology, interhospital hernia repair, and 5G-assisted spine fixation.^11^, ^12^, ^13^, ^14^ Notably, our 5 G packet loss was negligible (median 0.0%) and did not differ significantly from wired sessions (*p* = 0.096), indicating that the private 5 G configuration preserved data integrity despite the added wireless hop.

Task success ranged from 82.1% (Task 2, dissection) to 100% (Task 1, object manipulation), with all failures and partial successes occurring exclusively during wired sessions. While this pattern might suggest superior performance under 5 G, the finding is most parsimoniously explained by non-randomized allocation and temporal confounding: wired sessions spanned three days with 23 participants of varying experience levels and surgical specialties, whereas the five 5 G sessions were concentrated in a single afternoon period on Day 3. Surgeons in the 5 G cohort may have benefited from institutional familiarity with the platform accumulated during prior study days, and the smaller sample reduces the probability of capturing outlier failures. The non-significant Fisher exact tests (Task 2: *p* = 0.55; Task 3: *p* = 1.00) appropriately reflect insufficient power, and the observed pattern should not be interpreted as evidence of 5 G superiority. Completion times were consistent with the expected complexity of each task (median 26 s for manipulation, 4.5 min for dissection, 8.5 min for suturing) and comparable across network conditions, although formal statistical comparisons were limited by the unbalanced sample.

Surgeon perceptions were predominantly favorable and merit further examination. The vast majority (96.4%) considered the system suitable for clinical use, and 78.6% assigned the maximum safety rating (5/5), with the remaining 21.4% assigning 4/5 ‒ indicating that no participant considered the system unsafe. Four participants (14.3%) reported transient loss of control during operation, with three recovering fully, and only one (3.6%) reported errors or near-miss events. These rates are reassuring for a first-use dry lab scenario with a novel platform and align with the favorable safety profiles reported in recent 5 G clinical trials and multicenter experiences.^5^, ^6^, ^7^, ^8^, ^9^, ^10^ Regarding complexity suitability, 67.9% of surgeons believed the system could support high-complexity procedures, while 32.1% considered it more appropriate for medium-complexity cases ‒ a distribution that likely reflects individual differences in risk tolerance rather than platform limitations, given the uniformly high safety ratings.

Analysis of mental workload via PCA revealed that a single general workload component (PC1) explained 51.3% of the variance in NASA-TLX subscale scores, with effort, mental demand, and frustration loading positively and performance loading inversely. This structure is consistent with the dimensional design of NASA-TLX.^3^ The second component (PC2, 20.3%) was driven primarily by temporal demand, suggesting that time pressure constituted a distinct source of workload beyond general task difficulty. Ordinal regression models found no significant associations between network type, connectivity metrics (latency, jitter), or robotic experience and perceived safety or procedure complexity (all *p* ≥ 0.27). While these null results are likely attributable to the limited sample size and low variance in outcomes ‒ nearly all safety ratings were 4 or 5 ‒ they also indicate that the higher latency in 5 G sessions did not produce a detectable shift in surgeon perception, consistent with the premise that 32.4 ms remains below the threshold of subjective noticeability for trained operators.

The SUS context adds a distinctive pragmatic dimension to these findings. To our knowledge, this is the first study reporting telesurgery feasibility data from within Brazil's public healthcare system. Demonstrating stable task performance across both a public wired link and a private 5 G segment between two academic hospitals (HCFMUSP and HU-USP, approximately 5 km apart) suggests that remote robotic workflows can be piloted within existing public infrastructure. This approach parallels the Chinese model of expanding surgical access through telesurgery, as described by Lan et al. and Tian et al., who outlined structured safety protocols and institutional experience with 5 G robotic gastrectomy.^15^^,^^16^ In a continental country marked by pronounced regional disparities in specialized surgical care, academic public hospitals may serve as initial testbeds for building operational protocols, regulatory pathways, and training curricula. As the field matures, expert consensus guidelines addressing network reliability, cybersecurity, and deployment safeguards as well as multi-year validation frameworks such as the five-year hinotori assessment in Japan provide essential references for developing SUS-specific implementation standards.^17^^,^^18^ The Toumai platform used in our study represents a relevant choice for this context: its multicentric experience and prospective trial data demonstrate operational stability across diverse specialties and network configurations at latency profiles comparable to those observed in our setting.^9^^,^^10^

Several limitations must be acknowledged. First, the study used short, standardized dry lab tasks rather than complete clinical procedures, limiting generalizability to operative conditions. Second, network allocation was non-randomized, with wired sessions spanning three days and 5 G restricted to a single afternoon, introducing potential confounding from temporal and fatigue factors. Third, the small 5 G sample (*n* = 5) substantially limits statistical power, as reflected in the non-significant Fisher exact and ordinal regression tests. Fourth, per-participant mean network metrics may mask transient latency or jitter spikes that could affect precision during critical maneuvers. Fifth, the private 5 G configuration used the institutional wired connection as backhaul to a local CPE gateway; while this isolates the wireless hop ‒ the segment most relevant to telesurgery latency ‒ it does not replicate a full end-to-end commercial or dedicated 5 G deployment with an independent core network and infrastructure. Sixth, this single-platform, single-city study may not be generalizable to longer distances, different network architectures, or other robotic systems. Seventh, the absence of objective performance metrics (e.g., instrument path length, economy of motion) beyond binary task success limits the sensitivity to detect subtle performance differences between conditions.

Future work should prioritize prospective randomized designs comparing network modalities with longer and more complex procedures, incorporating clinical endpoints such as complication rates and objective measures of operative precision. Continuous real-time network logging ‒ rather than session-level summaries ‒ would enable detection of transient instability events that may affect surgical safety. Multicenter trials across geographically diverse Brazilian sites are essential to assess performance over varying distances and network conditions within SUS. The development of operational thresholds for latency, jitter, and packet loss specific to SUS deployment would provide actionable benchmarks for regulatory and clinical decision-making. Finally, building on existing international guidelines, the establishment of a national framework for telesurgery safety, training, and credentialing is a necessary step toward the responsible introduction of remote robotic surgery in Brazil's public health system.^17^^,^^18^

## Conclusion

Robotic telesurgery within SUS infrastructure was feasible in a simulated environment. Although the private 5 G network exhibited higher latency and jitter than the wired connections, task completion and usability were preserved. These results support continued evaluation of telesurgery within SUS, including controlled clinical trials.

## Abbreviations

SUS, Sistema Único de Saúde; FMUSP, Faculdade de Medicina da Universidade de São Paulo; HU-USP, Hospital Universitário da Universidade de São Paulo; PROMIN, Centro de Treinamento em Procedimentos Minimamente Invasivos; CPE, Customer Premises Equipment; PCA, Principal Component Analysis, NASA-TLX-NASA, Task Load Index, QoS, Quality of Service, IQR, Interquartile Range.

## Data availability

All data are available within the text and associated tables.

## Declaration of generative AI use

Generative artificial intelligence tools were used during the preparation of this manuscript to assist with text drafting. All AI-generated content was critically reviewed, verified, and edited by the authors, who take full responsibility for the final content of the manuscript. The model used was Claude’s Opus 4.5.

## Authors’ contributions

Everson L. A. Artifon and Jose Pinhata Otoch served as general coordinators of the project and were responsible for overall study organization and supervision. Giovanni G. Cerri contributed to the articulation and institutional structuring of the project. Felipe Kfouri contributed to data collection, manuscript writing, and statistical analysis. Luiz P. Kowalski, William Nahas, Paulo Pego-Fernandes, Edmund C. Baracat, José Maria Soares Júnior, Ulysses Ribeiro Jr., Marcos Samano, Ricardo Terra, Pedro Nabuco, Marco A. V. Kulcsar, Gabriel dos Anjos, Eduardo Motta, Ricardo Zugaib Abdalla, Sergio Damous, and Alessandro Belon contributed to the organization and coordination of the surgical teams.

## Funding

The robotic platform was provided by the manufacturer at no cost for the purpose of this feasibility study. No additional external funding was received.

## Conflicts of interest

The authors declare no conflicts of interest.

## Appendix Supplementary materials

Image, application 1
