# Supplementary material for: Feasibility of robotic telesurgery over wired and private 5 G networks within Brazil’s public health system (SUS): a pilot study
Source: Clinics (Sao Paulo). 2026 Apr 2;81:100926. doi: 10.1016/j.clinsp.2026.100926 (PMC13084647; doi:10.1016/j.clinsp.2026.100926)
Supplement: Supplementary file 1 [file mmc1.pdf]

CLINICS-D-25-00376_Supplementary Material

Supplementary Table 1 Structured questionnaire domains and items used in the interview.

Demographics
Age;
Sex;
Specialty/subspecialty;
Years of surgical and robotic experience;
Approximate number of robotic cases in prior year;
Prior tele-mentoring experience;
Familiarity with robotic platforms.
Workload and clinical Feasibility
Mental demand: How much mental and perceptual activity was required? (Likert 1–5)
Temporal demand: How much time pressure did you feel while performing the task? (Likert 1–5)
Performance: How well do you think you achieved the goals of the task? (Likert 1–5)
Effort: How much mental and physical effort was required to achieve your level of performance? (Likert 1–5)
Frustration level: How insecure, irritated, stressed, or frustrated did you feel during the task? (Likert 1–5)
Perceived video quality (Likert 1–5)
Perceived latency (Likert 1–5)
Perceived occurrence of freezes or frame drops (Likert 1–5)
Audio quality and synchronization (Likert 1–5)
Control responsiveness (latency, smoothness) (Likert 1–5)
Precision and dexterity achievable with the system compared with conventional robotic systems (Likert 1–5)
Perceived loss of control or video? (Yes/No)
Do you believe that errors or near-misses may have occurred due to the telesurgery system? (Yes/No)
Perceived level of safety when performing the task (Likert 1–5)
Acceptability of the current latency for different types of procedures (single choice: low, medium, high)
Effectiveness of communication with the on-site team (Likert 1–5)
Ability to regain control in the event of a technical failure (Yes / No / Partial / Not applicable)
Suitability for clinical use (yes/no)

Supplementary Table 2 Task outcome counts stratified by network type.

Task	Network	Success	Partial Success	Failure	Total
Task 1 (Peg transfer)	Wired	23	0	0	23
	5G	5	0	0	5
	Total	28	0	0	28
Task 2 (Ring transfer)	Wired	18	0	5	23
	5G	5	0	0	5
	Total	23	0	5	28
Task 3 (Suture/ knot)	Wired	20	3	0	23
	5G	5	0	0	5
	Total	25	3	0	28

**Outcome categories**	
Success	Task completed within time limit without critical errors
Partial Success	Task completed with notable difficulty or minor errors
Failure	Task not completed or abandoned

Notes:

Total sessions: 28 (23 wired, 5 private 5G).

Fisher's exact test results for each task are reported in the main text.
